# Solvent Front Position Extraction with Semi-Automatic Device as a Powerful Sample Preparation Procedure Prior to Quantitative Instrumental Analysis

**DOI:** 10.3390/molecules24071358

**Published:** 2019-04-06

**Authors:** Anna Klimek-Turek, Kamila Jaglińska, Magdalena Imbierowicz, Tadeusz Henryk Dzido

**Affiliations:** Physical Chemistry Department, Medical University of Lublin, Chodźki 4A, 20-093 Lublin, Poland; kamila.skop@umlub.pl (K.J.); magdalenaimbierowiczumlub@gmail.com (M.I.); tadeusz.dzido@umlub.pl (T.H.D.)

**Keywords:** sample preparation with TLC/HPTLC, solvent front position extraction, solvent delivery with a moving pipette, automation, LC–MS/MS

## Abstract

The new prototype device is applied to the Solvent Front Position Extraction (SFPE) sample preparation procedure. The mobile phase is deposited onto the chromatographic plate adsorbent layer by the pipette, which is moved, according to programmed movement path, by a 3D printer mechanism. The application of the prototype device to SFPE procedure leads to the increased repeatability of the results and significant reduction of the analysis time in comparison to the classical procedure of chromatogram development. Additionally, the new equipment allows use procedures that are not possible to run using the classic chromatogram development. In this paper, the results of manual and semi-automatic sample preparation with SFPE are compared and the possible application of this prototype device is discussed.

## 1. Introduction

Sample preparation is an integral part of any analytical method, influences all subsequent steps of the analysis, and has a relevant impact on the precision and accuracy of the results [1]. Unfortunately, it is still the most labor-intensive step of the analytical procedure, consuming two-thirds of the total analysis time [2,3]. This is in strong contrast to modern, fast chromatographic methods, mass spectrometric detection, and makes sample preparation a limiting step of the analysis. In the light of these considerations, it is not surprising that there is a growing interest in the automation of this process [4]. According to the result of a survey carried out among analysists, the percentage of respondents using automated sample preparation jumped significantly, from 29% in 2013 to 39% in 2015 [4]. This is perfectly understandable if one takes into account that the application of automation sample preparation techniques reduces sample manipulation, and provides high recovery, throughput, and speed of analysis [4].

Many automated systems operate in off-line mode, when the sample preparation procedure is independent of further analysis [5]. In recent years, there has been a significant increase of interest in on-line processing techniques, where both sample preparation and instrumental analysis operate as a single system [6]. On-line mode allows the determination of target compounds automatically in a fast and efficient way. The online coupling of sample preparation techniques with LC began from the connection of solid phase extraction (SPE) to liquid chromatography (LC) [7]. Recently, a variety of online sample preparation techniques have been emerging, such as solid phase microextraction (SPME) [8,9,10], microextraction in packed syringe (MEPS) [11,12], stir bar sorptive extraction (SBSE) [13], liquid phase microextraction (LPME) [14], accelerated solvent extraction, (ASE) [15], microdialysis (MD) [16], microwave assisted extraction (MAE) [17], supercritical fluids extraction (SFE) [18], and other modern technologies which can overcome weaknesess and shortcomings of the traditional techniques of sample preparation.

Undoubtedly, among the online liquid sample preparation techniques, the column-based SPE is the most commonly used one [19]. In this technique, the one mobile phase is used to introduce the sample into the first column called the ‘extraction column’ and then the components are washed out to the second column, the so-called analytical one, using a different mobile phase. The direction of the mobile phase flow through the extraction and analytical column is the same [20]. The other technique uses a back-flush mode. Then the sample solution is introduced in the extraction column and the analytes are retained in the head of it. In the subsequent stage, the analytes are washed out to the analytical column by a mobile phase flowing in the opposite direction with respect to the flow during sample introduction into the extraction column [21,22].

The major advantage of SPME technique is its relatively easy automation. The technique integrates sampling, extraction, preconcentration, and injection into one analytical system, which is simple, efficient, and solvent-free [23].

Micro-extraction packed sorbent is also the kind of miniaturization of conventional SPE from milliliter to microliter bed volumes [24]. The manipulation of the small volumes is realized with a precision gas-tight syringe. The automated online MEPS-LC can be achieved by connecting the syringe to an autosampler [25].

A quite different approach to the sample preparation procedure is used in Dried Blood Spot (DBS) analysis [26,27]. This method has recently gained attention in bioanalytical laboratories.

In this method, after application to special cards, the samples are dried, collected, transported, and stored in a dry form. In order to enable analysis, each card is extracted for the isolation of target analytes, and the obtained solution typically undergoes LC–MS/MS analysis. Traditional DBS techniques are highly labor-intensive, require manual card punching, extracting the sample from the blot paper, centrifugation, and then transfer of the supernatant to a vial for further analysis. Fortunately, in recent years automated DBS card extraction and analysis systems have been developed in order to meet or exceed the performance of manual methods for sensitivity, accuracy, reproducibility, and precision in the analysis [28].

In our previous papers, we presented a solvent front position extraction (SFPE) technique, which is based on planar solid phase extraction clean-up concept for pesticide residue analysis developed by Oellig and Schwack [29,30]. In the papers thin-layer chromatography was used to sample preparation for the determination of solutes followed by HPLC (High Performance Liquid Chromatography) [31] or MS (Mass Spectrometry) analysis [32]. SFPE procedure was used to separate substances of interest (acebutolol, aminophenazone, acetaminophen, caffeine, theophylline, tramadol, ciprofloxacin, acetylsalicylic acid) and their internal standard (acetanilide) from other matrix components (bovine serum) and to form a single spot/zone containing them at the solvent front position on a chromatographic plate. We obtained very promising results. Some disadvantages of the presented technique (e.g., time-consuming, relatively high values of percent relative standard deviation (%RSD)) were mostly due to a large number of manual operations. In this work, we present the next stage of our research which makes feasible the semi-automation of the proposed technique. We propose a new prototype device which is applied to SFPE procedure. A part of manual operations are replaced by using prototype device; additionally, new equipment allow the use of procedures that are not possible to run using the classic chromatogram development. We compare the results of manual and semi-automatic sample preparation with SFPE and discuss the prospects for the approach mentioned.

## 2. Results and Discussion

In our previous articles [31,32], we proposed a new approach to biological sample preparation for instrumental analysis. The results obtained using the presented SFPE procedure were very promising. 

However, the number of manual operations needed to perform the SFPE procedure seems to be the main limitation. The necessity of cutting the plates into fragments corresponding to the sample track followed by chromatogram development from two opposite sides along the short edge of the plate towards its middle considerably prolongs the time of the procedure and makes it quite tedious. Unfortunately, in manual execution of SFPE procedure, this stage has to be performed because it eliminates the influence of the radial chromatography effect (occurring during the sample application) on spreading of substances of interest in their spots in the start line and in the solvent front position. Therefore, in order to make this stage more user-friendly, a prototype semiautomatic device for delivering the eluent to the chromatographic plate has been designed. Details of this prototype device construction are presented in the Experimental section.

The new prototype allows delivering the mobile phase at a controlled velocity to the chromatographic plate by the pipette moved slightly above the adsorbent layer. The similar type of device based on the 3D printer’s mechanism has been reported by our group to test separation efficiency of substances on chromatographic plate with adsorbent layer face down [33]. The equipment presented in the paper is somewhat different in construction and is especially adapted to sample preparation with SFPE. First of all, the mobile phase is delivered to the adsorbent layer face up. This makes it so that any horizontal TLC chamber can be easily adapted to perform SFPE, i.e., by production a slit in glass cover plate of the chamber. In the mentioned paper, the horizontal chromatographic chamber was equipped with a slit (a gap for pipette movement) produced in its bottom. In the case of the proposed prototype, the chromatographic plate may be placed in a standard horizontal chromatographic chamber. Additionally, the pipette is to move along three axes at various speeds, which allows delivery of the mobile phase to any position onto the chromatographic plate and development of chromatograms in any desired direction. This property of the device is very essential for SFPE, especially at the stage of substance zone narrowing, which is documented and discussed in the paper.

### 2.1. Preliminary Research

As was described in the previous paper [32], the accuracy and precision of the SFPE technique are based on the assumption that both sample components and the internal standard after chromatograms developments were evenly focused (preconcentrated) in the zone of the solvent front. In order to fulfil that requirement, the substance (or substances) and the internal standard should show the value of the coefficient ^n^R_F_ (relative distance migration of a solute after the n-th development) equal to at least 0.99. Therefore, to fulfil this requirement, the number of chromatogram developments has to be determined before application of SFPE to sample preparation.

**Figure 1:**
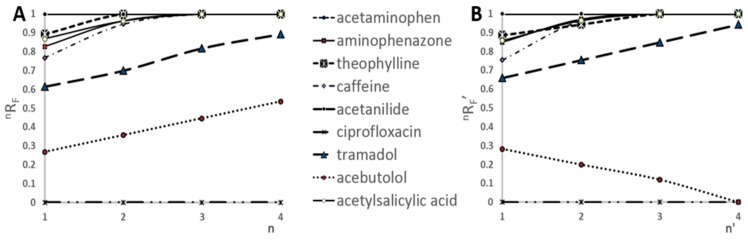
(**A**) ^n^R_F_ of solutes vs. the number of developments. (**B**) ^n^R_F_’ of solutes vs. the number of developments; biological matrix sample; chromatographic plate: HPTLC Silica gel 60 F254, mobile phase: methanol.

The relative migration distance of all substances increases according to number of developments when the mobile phase is delivered below the start line. This is valid if R_F_ (retardation factor) of substance after the first development is greater than 0. The dependence of ^n^R_F_ vs. the number of developments using prototype device is presented in Figure 1A. The ^n^R_F_ refers to the experiments, when each development of the chromatogram was performed by delivering the mobile phase onto the adsorbent layer of chromatographic plate by pipette moved along the path located between the lower edge of the chromatographic plate and the start line—path position Y1 (Figure 8). One can see that the ^n^R_F_ increases with each chromatogram development for all substances except ciprofloxacin (that stays in the start line in this chromatographic system applied). Similar results were obtained by developing the chromatograms in a conventional horizontal DS chamber [32]. As was mentioned above, the prototype device gives the important advantage of developing the chromatogram from any chosen solvent entry position on the adsorbent layer. This feature was used during the experiments. The first chromatogram development was carried out as described above, however, the subsequent developments were caried out by delivering the mobile phase onto the adsorbent layer of the chromatographic plate by the pipette moved along the path parallel to the *X*-axis at path position Y2 (Figure 8). For such chromatogram development the relative migration distance have been denoted as ^n^R_F_’. In this case ^n^R_F_’ of the substances increases with each consecutive chromatogram development too. However, it is applied to the substances tramadol, acetylsalicylic acid, aminophenazone, caffeine, theophylline, acetaminophen, acetanilide, which showed zones located above the path position Y2. On the other side ^n^R_F_’ of acebutol decreases with each subsequent development because its zone was below the path position Y2 of the moving pipette.

The delivering of the mobile phase to any position on the chromatographic plate is very important for SFPE technique especially in case of samples with reach matrix because it prevents elution of the interfering compounds to the solvent front zone and facilitates preconcentration of components of interest in this zone.

It also can significantly shorten the time of analysis. It is not necessary to multiply develop chromatogram from the bottom edge of the plate, but from the path position of the moving pipette located above the zones of unwanted matrix substances.

Based on the results presented in Figure 1B, the time necessary for four-fold chromatogram development, when the path position of the the mobile phase delivery onto the adsorbent layer of the chromatographic plate was Y2 (above the starting spots), had been reduced by 40% compared to the classical four-fold chromatogram development, while keeping the obtained results on similar level of accuracy.

The main goal of our research was to determine if the prototype device would have been streamlined the process of SFPE sample preparation. Therefore, we did not focus on the determination of all substances in the sample, but rather on presenting this possibility by the prototype device in this regard. Based on the results presented in Figure 1, it can be concluded that the minimum number of developments, that guarantees the values ^n^R_F_ (or ^n^R_F_′) higher or equal to 0.99 for at least six of the nine analytes, is four. Consequently, in the following experiments, all chromatograms were developed four times. It means that six components (acetylsalicylic acid, aminophenazone, caffeine, theophylline, acetaminophen, acetanilide) of nine in the test mixture are used for presentation of quantitation with semiautomatic procedure. In the next paper, we will show an optimization procedure for quantitation of all mixture components investigated in the paper.

### 2.2. Quantitation Result with SFPE Procedure

#### 2.2.1. Methanolic Sample

**Figure 2:**
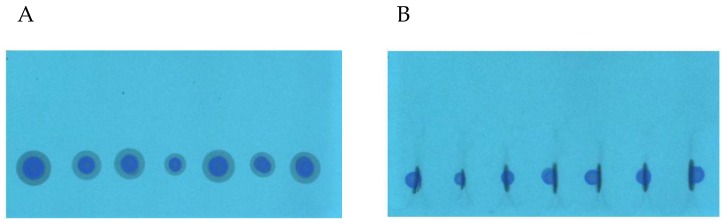
The mixture (methanol sample) chromatograms: (**A**) after sample application; (**B**) after spot narrowing procedure. HPTLC Silica gel 60 F254 (Merck), mobile phase: methanol.

During the SFPE procedure the samples were applied as a drops on the chromatographic plate using automatic pipette. This method of sample application is convenient because it does not require advanced equipment and can also be carried out outside the laboratory. It is also an ideal choice for samples which cannot be applied to the chromatographic plate by an automatic applicator because of their viscosity or the amount of contaminants. Unfortunately, such a method of sample application results in radial chromatography, especially when the substances of interest differ in retention. In the previous study [32], we showed that in order to obtain appropriate quantification results the effect of radial chromatography occurring at the sample application stage (Figure 2A) should be eliminated. The procedure of this effect elimination using the prototype device is presented in the Experimental section as the narrowing of the starting spots. The start spots after the narrowing procedure are presented in Figure 2B. One can see that the zones of the starting spot are considerably narrowed (except the zone of ciprofloxacin, because these substances do not migrate in the applied chromatographic system). The use of the prototype device allowed narrowing of the substance zones of many samples applied on the plate, with minimal involvement of manual operations. In the previous paper, each sample track had to be cut into a single chromatographic plate strip to manually perform the narrowing procedure.

After the narrowing procedure (S), chromatograms were developed using SPK3 mode and then the solutes were extracted from solvent front position with methanol using the CAMAG TLC–MS interface. The results of the substance determination are presented in Table 1. %RSD and the percentage difference of the results obtained by the dLCMS method and by the LCMS combined with the SFPE technique exceeds 5% for tramadol and aminophenazone only. The use of the device improved the results for the theophylline, caffeine, and acetylsalicylic acid [32]. Unfortunately, as in the previous research, there was a problem with tramadol and aminophenazone determination. Less satisfactory results obtained for aminophenazone are mostly due to the specific substance properties—probably the analyte decomposes during the analysis. This substance is “air and light sensitive” [34] what can explain the problem. The similar problem with tramadol also occurred in our previous work [32] and could be solved by changing the pH of the mobile phase [35].

It is particularly noteworthy that the time of implementing the SFPE technique, especially the stage of narrowing the start zones, is considerably shortened. The narrowing time is about 10 s per sample, while in the case of manual execution of this procedure it is about 40 s (omitting the time needed to dry the plates between the two consecutive developments and cutting the plates).

The ‘manual’ method of the narrowing starting zone used in the our previous research prolonged the SFPE procedure not only at this but at developing chromatograms stage as well. In such a case, chromatograms of each applied sample had to be developed separately. For seven sample analyses, the development time was extended seven times in comparison with the situation when all seven samples are developed simultaneously on a single plate.

Of course, it is possible to put the cut pieces of the chromatographic plate in several chambers and develop chromatograms at the same time, but this requires careful keeping track of several solvent fronts at the same time, and thus it requires a lot of laboratory personnel involvement.

Last but not least—using prototype devices for narrowing starting spots and chromatograms development will can make the SFPE procedure fully-automatic.

#### 2.2.2. Serum Samples

**Figure 3:**
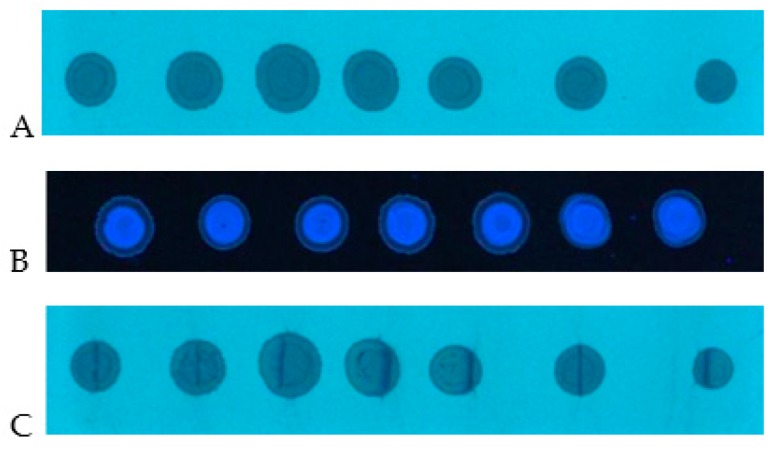
Mixture (serum sample) starting spots: (**A**) after sample application, λ = 254 nm; (**B**) after sample application, λ = 366 nm; (**C**) after starting spot narrowing, λ = 254 nm. HPTLC Silica gel 60 F254 (Merck), mobile phase: methanol.

The next step was the application of prototype device to the SFPE procedure for substance quantitation in a sample with a biological matrix such as bovine serum. Figure 3A shows the spots formed after the application of serum on the chromatography plate (applied volume was 10 μL per spot). At this stage, contrary to the methanol samples, the effect of radial chromatography is less visible, what is probably suppressed by matrix components. This effect, however, occurs, which is more clearly seen at the wavelength 366 nm (Figure 3B). This allowed us to suppose that quantitation results obtained by any conventional chromatogram development, without narrowing the start zone, would not have been accurate.

**Figure 4:**
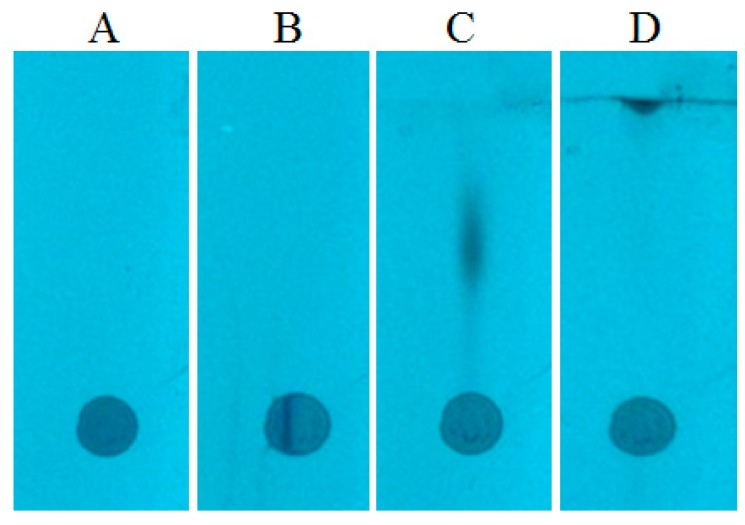
The mixture (serum sample) chromatograms: (**A**) after sample application; (**B**) after starting spot narrowing; (**C**) after first development; (**D**) after fourth development. Stationary phase: HPTLC Silica gel 60 F254. (Merck), mobile phase: methanol.

In order to eliminate the effect of radial chromatography, the prototype device was used for narrowing the starting spot (as in case of the methanol sample discussed above). The spots after the narrowing procedure are presented in Figure 3C. One can see that the substances are gathered in a narrow zone. Consequently, after the chromatogram development, the substances are concentrated in a small area (Figure 4D), which could be almost completely covered by the head of TLC–MS interface.

**Figure 5:**
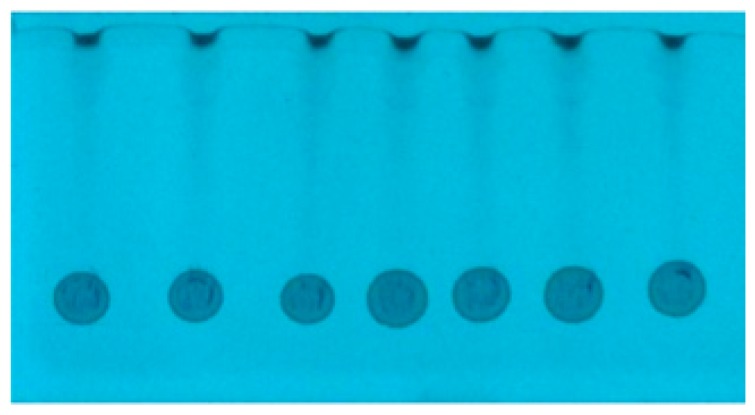
The serum sample chromatograms after development using SPK3 procedure; stationary phase. HPTLC Silica gel 60 F254 (Merck), mobile phase: methanol.

The chromatograms were developed using the SPK3 mode. The obtained chromatograms are presented in Figure 5. The percentage difference of the results obtained by the dLCMS method and by the LCMS combined with the SFPE procedure are lower than 5%. However, this value is considerably exceeded for tramadol and aminophenazone only (Table 1). The explanation for this phenomena is discussed above.

It should be underlined that the determination results are far better in comparison to our previous work [32]. The %RSD decreased below 5% for caffeine, theophylline and acetylsalicylic acid. It is worth to stress that the number of chromatogram developments in this work is four, while in the previous work it was 6. Using the prototype machine we managed to get a %RSD value below 5 for four substances, while in the previous work only for one (acetaminophen).

The advantages of the prototype device are analogous to those discussed in the case of methanol samples. Additionally, the possibility of delivering mobile phase to any place on the chromatographic plate allows chromatogram development above the application zone. It is very important in the case of samples with reach matrix, when the repeated wetting of matrix zones leads to the elution of the interfering compounds to the solvent front zone.

Moreover, it should be mentioned that the ‘edge’ effect (encountered in the classical chromatogram developments) did not occur when the prototype device was used at controlled velocity of the mobile phase. This is a very desirable feature, especially if the SFPE technique will be fully automated.

## 3. Materials and Method

### 3.1. Materials and Reagents

Chromatographic plates, HPTLC Silica gel 60 F254, 10 × 20 cm, were supplied by Merck (Darmstadt, Germany). Methanol, water and formic acid LC–MS grade were purchased from Merck (Darmstadt, Germany). Acetaminophen, acetanilide, aminophenazone, caffeine, acetylsalicylic acid, and theophylline were purchased from Sigma–Aldrich (St. Louis, MO, USA). Acetobutolol was purchased from Biomedicals, USA (Santa Ana, CA, USA), ciprofloxacin from Sreepathi Pharmaceutical Ldt. India (Telangana, India), Tramadol from Inogent Laboratories, India (Telengana). Bovine serum was purchased from Biomed (Lublin, Poland).

### 3.2. Preparation of Internal Standard and Analyte Solutions

The stock solutions were prepared by dissolving proper amounts of each substance in methanol and stored in a refrigerator at 4 °C. Solutions of the substances mentioned were prepared by adding 10 µL of each stock solution to 1 mL of serum and methanol and finally were in the range of substances concentrations typically found in real blood/serum samples (Table 2).

### 3.3. HPTLC Plate Preparation

HPTLC Silica gel 60 F25410 × 20 cm plates were cut into desired sizes using TLC plate cutter (CAMAG, Wilmington, NC, USA). Before chromatogram development, the plates were washed by immersion in methanol for 1 min. Afterward the plates were dried in the air and activated in an oven at 105–110 °C for 15 min.

### 3.4. Application of the Samples on the HPTLC Plate

The samples were applied as small single drops on the chromatographic plate using an automatic pipette (PZ HTL S.A., Warszawa, Poland). The volume of drop was about 10 µL. 

### 3.5. Prototype Device for Semiautomatic Sample Preparation Using SFPE Procedure

**Figure 6:**
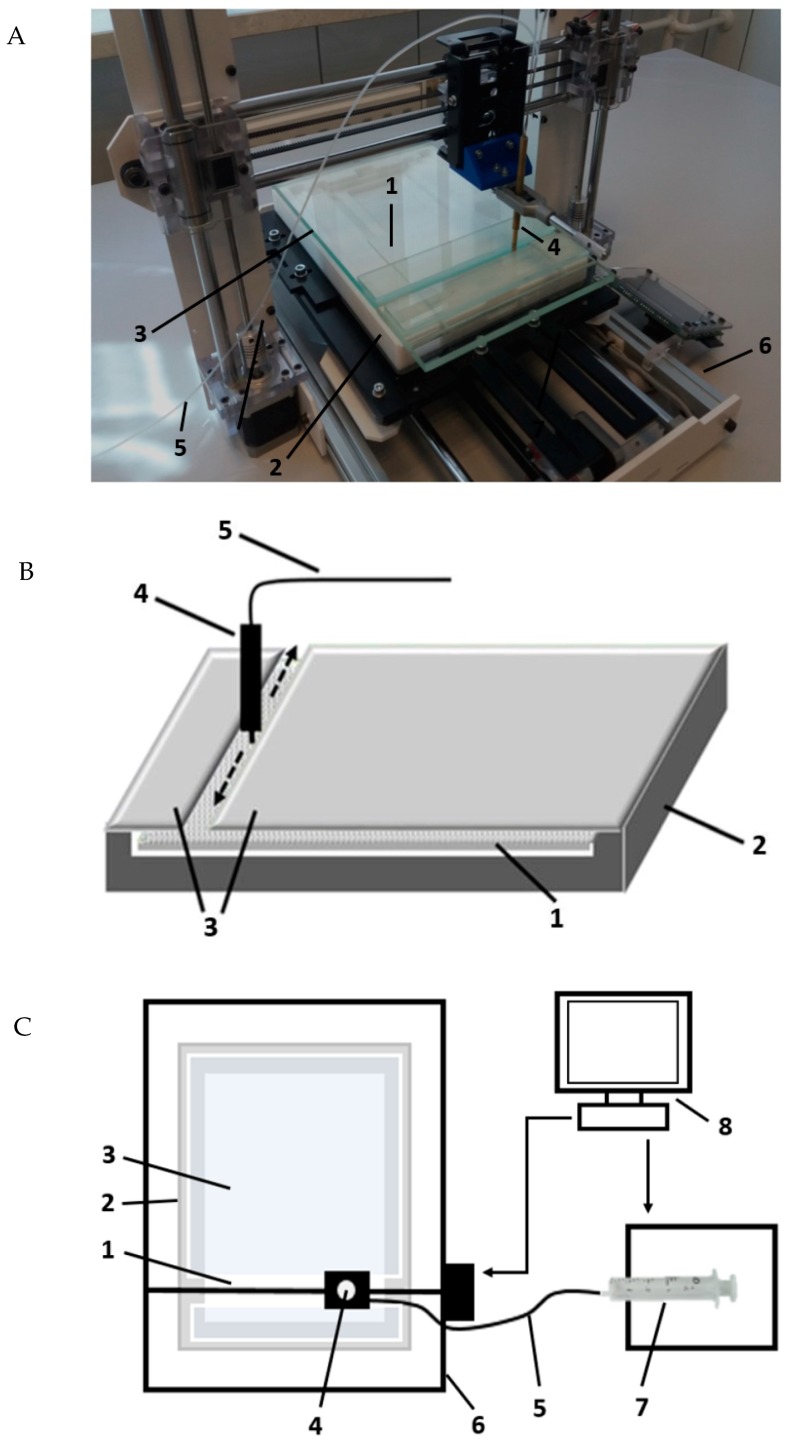
(**A**) The picture of the prototype device. (**B**) Axonometric view of the horizontal chamber with moving pipette. (**C**) Conceptual view of the whole device, 1—chromatographic plate, 2—Teflon horizontal TLC chamber, 3—cover glass plate, 4—moving pipette, 5—capillary, 6—3D machine, 7—syringe pump, 8—computer.

The experiments were performed using a new prototype device presented in Figure 6. The chromatographic plate (1) was placed in the horizontal Teflon chamber (2) with the adsorbent layer face-up (3). Then, the mobile phase was provided onto the adsorbent layer of the chromatographic plate by the pipette (4), which was moved, according to a programmed movement path, by 3D printer mechanism (6) controlled by computer (8). The tip of pipette was in close contiguity to the adsorbent layer (0.15 mm), not touching it. The pipette was equipped with the capillary (5) and combined with syringe pumps (7) (TYD02-01 Laboratory Syringe Pump, Leadfluid, Baoding, Heibei, China).

The pipette is able to move in three axes at a variety of speeds, which can deliver the mobile phase to any place on the chromatographic plate and develop chromatograms in any desired direction. When the chromatograms were developed, the chromatographic plate was covered with a glass plate (3).

### 3.6. Narrowing Procedure of the Sample Spots Applied on the Start Line

**Figure 7:**
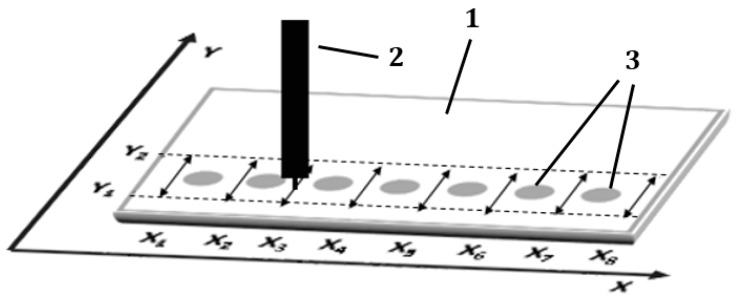
Scheme of the moving pipette paths represented by double-sided arrows for the narrowing procedure of the sample spots applied on the start line. 1—chromatography plate; 2—moving pipette; 3—sample spots applied on the chromatographic plate.

Single drops of sample solutions were spotted on the start line of the chromatographic plate. The plate was left in the air for 10 min to dry the sample spots. Then the starting sample spots were subjected to narrowing procedure, i.e., elimination of radial chromatography effect generated during sample application. During this procedure, the mobile phase solution was delivered to the chromatographic plate with the pipette moved by the 3D machine along eight paths each perpendicular to the *X*-axis and located between starting spots (see Figure 7). The length of these paths was determined by Y1 = 5 mm and Y2 = 25 mm, measured from the lower edge of the chromatographic plate, which were the turning points of the pipette’s movement. 

In each of the eight paths (X_1_ … X_8_), the pipette moved eight cycles of “Y1-Y2-Y1”. The speed of the pipette movement was 2000 mm/min and velocity of the mobile phase delivery to the adsorbent layer was 5 mL/h. When the procedure above for the all paths was completed the plate was dried in the air for 10 min.

### 3.7. Planar Chromatogram Development

**Figure 8:**
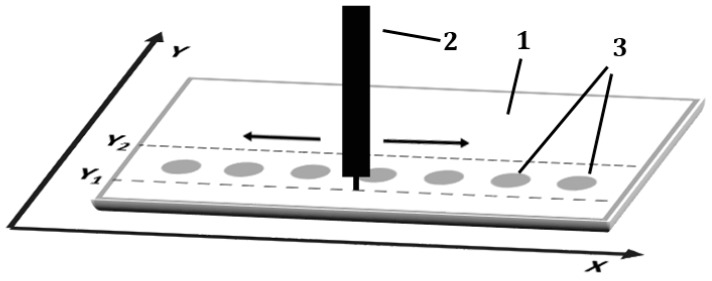
Scheme of the paths represented by the dashed lines for delivering the mobile phase solution to the adsorbent layer with moving pipette driven by 3D machine. 1—chromatography plate; 2—moving pipette; 3—sample spots applied on the chromatographic plate.

In each development variant, the pipette tip was moved over the entire width of the chromatography plate (back and forth, parallel to the *X*-axis and perpendicular to the *Y*-axis) at constant speed of 2000 mm/min (Figure 8). The distance of the pipette tip from the surface of the adsorbent layer was 0.15mm. The speed of the mobile phase solution delivery to the adsorbent layer was equal to 5 mL/h.

#### Variants of Sample Preparation for Quantitation

• SPK3 variant

Single drops of sample solutions were spotted 15 mm apart from each other on the start line of the chromatographic plates. The start line was located at the distance 15 mm from the chromatographic plate edge. After drying of the spots obtained (10 min) narrowing procedure described above was performed. Then the chromatograms were developed one time in horizontal Teflon chamber using pipette parallel moved to the *X*-axis at *Y* = 10 mm from the lower edge of the chromatographic plate. Distance of chromatogram development, measured from start line, was equal to 35mm. Next the chromatograms were developed three times in the chamber applying pipette parallel moved to the *X*-axis at *Y* = 25 mm from the edge of the chromatographic plate. In this case, the distance of movement of the mobile phase front was equal to 20 mm. After each development the plates were dried in the air for 10 min. Then the plate was subjected to extraction of the components of interest from the final position of the mobile phase front. The extraction was performed by using the TLC–MS interface from CAMAG, extrahent: methanol. The obtained sample solutions were injected (10 µL) into HPLC column of Agilent Zorbax Eclipse Plus C18 (Santa Clara, CA, USA) for their quantitation.

• dLCMS variant

Substances were determined by LC–MS/MS (Liquid Chromatography Tandem-Mass Spectrometry, Agilent, Santa Clara, CA, USA) technique without SFPE preparation procedure—i.e., the standard solution was directly injected to HPLC column of the Agilent 1290 chromatograph. 

### 3.8. Instrumentation

Thin-layer chromatography experiments were performed using a prototype of 3D machine with controlled eluent flow (Lublin, Boland, Infinum 3D) equipped with the horizontal Teflon chamber for TLC. For plate image documentation the TLC Visualizer, CAMAG (Muttenz, Switzerland), was used. The CAMAG TLC–MS interface, connected with Agilent 1260 Infinity isocratic pump, was used for extraction of the substances from the chromatographic plates. The Agilent1290 Infinity LC System (Santa Clara, CA, USA) connected with Agilent 6460 Triple Quadrupole was used for the LC–MS experiments. The chromatography was performed with the Zorbax Eclipse Plus-C18 column (4.6 × 100 mm, 3.5 µm, Agilent, Santa Clara, CA, USA). Mobile phase: A: 0.1% formic acid in water B: 0.1% formic acid in methanol. The gradient program was as follows: 0 min: 95% A, 5% B; 2 min: 75% A, 25% B; 9 min: 5% A, 95% B; 10min: 95% A, 5% B; 11 min: 95% A, 5% B. Data were acquired in the positive- and negative-ion modes (multiple-reaction monitoring mode) with electrospray probe voltages of 3500 V. The nebulizer gas setting was 15 psi. The ion source was operated at a temperature of 350 °C and a drying gas setting of 7 L/min. 

## 4. Conclusions

In this paper, it has been shown that the application of the prototype device to SFPE procedure leads to increased repeatability of results and significant reduction of analysis time in comparison to the classical procedure of chromatogram development.

The pipette of the prototype device is able to move along three axes at different speeds, which allows delivery of the mobile phase to any position on the chromatographic plate and development of the chromatograms in any direction. This advantage can narrow the substance start zones for many samples on one chromatographic plate without the necessity of cutting the plate into single track strips.

This device enables the mobile phase delivery to any position on the chromatographic plate. This feature is very advantageous for samples with a reach matrix, because it prevents interfering compounds from reaching the solvent front zone during SFPE.

The problem with determination of some substances in the paper could be solved by changing the pH of the mobile phase. This is preliminarily confirmed by our nonpublished investigations. The investigations are under development by our group and we hope to submit such results for publication in the near feature.

The presented results undoubtedly constitute a step in the process of full automation of the sample preparation by SFPE technique.

## Figures and Tables

**Table 1:** The values of relative standard deviation (%RSD) of average values (*n* = 7) of substance/acetanilide peak area ratios, the relative percentage difference between the results obtained by direct LCMS analysis and by LCMS analysis followed substances and internal standard extraction from final solvent front position by methanol with TLC–MS Interface (SFPE procedure). dLCMS and Mx are the average values of the substance/acetanilide peak area ratio obtained by direct LCMS and by LCMS analysis followed the extraction from final solvent front position by methanol with TLC–MS Interface (SFPE procedure), respectively. Chromatographic plate HPTLC Silica gel 60 F254.

	Methanolic Sample		Serum Sample	
	%RSD	100(Mx-dLCMS)/dLCMS	%RSD	100(Mx-dLCMS)/dLCMS
Tramadol	44.35	−74.9	24.65	−27.2
Acetylsalicylic acid	3.12	3.01	4.76	3.21
Aminophenazone	53.98	−51.24	24.92	−32.09
Caffeine	3.17	4.47	3.29	0.78
Theophylline	4.96	2.93	4.98	−3.15
Acetaminophen	4.52	4.03	4.98	−2.85

**Table 2:** Final concentration of substances in sample.

Substance	mg/L
Acebutolol	20.6
Ciprofloxacin	8.0
Tramadol	15.8
Acetylsalicylic acid	141.0
Aminophenazone	15.2
Caffeine	20.2
Theophylline	20.3
Acetaminophen	15.1
Acetanilide	15.2
